# *Giardia duodenalis* Infections in Humans and Other Animals in China

**DOI:** 10.3389/fmicb.2017.02004

**Published:** 2017-10-13

**Authors:** Junqiang Li, Haiyan Wang, Rongjun Wang, Longxian Zhang

**Affiliations:** College of Animal Science and Veterinary Medicine, Henan Agricultural University, Zhengzhou, China

**Keywords:** *G. duodenalis*, humans, animals, prevalence, assemblage, multilocus sequence typing, China, zoonotic

## Abstract

*Giardia duodenalis* is an important zoonotic pathogen in both public and veterinary health, and has been genotyped into at least eight assemblages (A–H), each with a distinct host range. In recent years, this intestinal protozoan parasite has been identified widely in humans and various other animals, and has even been recorded in environmental contaminants. Along with whole genome sequencing of *G. duodenalis*, multilocus sequence typing is increasingly being used to characterize *G. duodenalis* isolates. Here, we review the epidemiology, genotyping, and subtyping of *G. duodenalis* from humans and a wide range of other animals, as well as from wastewater, in China.

## Introduction

*Giardia* is one of the most common intestinal parasites of both humans and a diverse range of other animals (Feng and Xiao, 2011). The parasite was first discovered by Antonie van Leeuwenhoek over 300 years ago (Dobell, 1920), and since then, six *Giardia* species have been described. Among them, *Giardia agilis, Giardia ardeae, Giardia psittaci, Giardia muris*, and *Giardia microti* infect animals ranging from amphibians to rodents and birds, whereas the broad range of hosts for *Giardia duodenalis* (syn. *Giardia intestinalis* and *Giardia lamblia*) includes humans and domestic, farmed, and wild animals (Monis et al., 2009; Feng and Xiao, 2011; Ryan and Cacciò, 2013). Giardiasis, which is caused by *Giardia duodenalis*, is an important zoonotic disease for both public and veterinary health (Ryan and Cacciò, 2013).

The *G. duodenalis* life cycle is simple in that it comprises rapidly multiplying trophozoites (attached to intestinal epithelial cells) and cysts that are resistant to environmental degradation, which are excreted with feces and transmitted onwards via the fecal-oral route (Lv et al., 2013; Einarsson et al., 2016). *G. duodenalis* has long been considered to reproduce asexually by simple binary fission, but there is increasing evidence from epidemiological, molecular genetics, and whole genome sequencing studies that *Giardia* is capable of sexual reproduction (Cooper et al., 2007; Morrison et al., 2007; Poxleitner et al., 2008; Nolan et al., 2010; Gabín-García et al., 2017).

Molecular biological analysis of *G. duodenalis* has benefited from understanding the taxonomy, population genetics, and epidemiology of this pathogen, and such studies are essential to the effective control of giardiasis in clinical practice (Cacciò and Ryan, 2008). In terms of its genetic variation, *G. duodenalis* isolates genotypically fall into one of at least eight assemblages (A–H), each of which have a distinct host range (Cacciò and Ryan, 2008; Sprong et al., 2009; Feng and Xiao, 2011; Ryan and Cacciò, 2013). There is also genetic diversity within these assemblages. For example, sub-assemblages AI, AII, and AIII fall within assemblage A (Feng and Xiao, 2011) and BIII and BIV form assemblage B (Monis et al., 2003), while various sub-assemblages form assemblage E (Zhang et al., 2012c).

Multilocus genotyping (MLG) analysis using both conserved (e.g., *ssrDNA, ef, h2b, h4*) and variable (e.g., *tpi, gdh, bg*) genes was originally used to assess the *G. duodenalis* assemblages (Wielinga et al., 2011). Nowadays, variable genes such as *tpi, gdh*, and *bg* are used to characterize *G. duodenalis* isolates from humans and other animals and determine the genotype or subtype. These analyses provide sufficient resolution to assess the disease burden arising from zoonotic transmission of the parasite (Cacciò et al., 2008; Sprong et al., 2009; Wang et al., 2014b; Wang H. et al., 2016). To date, four genetically distinct *G. duodenalis* isolates (WB, AI; AS175, AII; P15, E, and GS, B) have been studied genomically (Franzén et al., 2009; Jerlström-Hultqvist et al., 2010; Adam et al., 2013) and transcriptomically (Franzén et al., 2013). Differences between the genomic and transcriptomic profiling results may explain the differences observed in host preferences and clinical presentation of *G. duodenalis* infection (Franzén et al., 2009, 2013; Jerlström-Hultqvist et al., 2010; Adam et al., 2013).

Annually, 280 million people worldwide are estimated to have clinically diagnosable giardiasis (Feng and Xiao, 2011; Ryan and Cacciò, 2013; Einarsson et al., 2016; Squire and Ryan, 2017), and infection rates are higher in developing countries (Feng and Xiao, 2011; Ryan and Cacciò, 2013). Giardiasis is generally a self-limiting clinical illness characterized by watery diarrhea, abdominal cramps, bloating, weight loss, and malabsorption (Feng and Xiao, 2011; Einarsson et al., 2016). However, asymptomatic infections occur more frequently than symptomatic ones (Himsworth et al., 2010; Feng and Xiao, 2011; Ryan and Cacciò, 2013; Wegayehu et al., 2016). In China, approximately 28.5 million giardiasis cases are estimated to occur in humans per year (Feng and Xiao, 2011), although the true incidence is likely underestimated as there are many undetected and/or unreported cases. In recent years, *G. duodenalis* has been identified in humans, non-human primates (NHPs), ruminants, companion animals, domestic animals, wildlife, and even in the environment in China (Liu et al., 2011; Wang et al., 2011, 2014b; Li N. et al., 2012; Zhang et al., 2012c; Liu A. et al., 2014; Liu H. et al., 2014; Li J. et al., 2015; Qi et al., 2016a,b; Wang H. et al., 2016). Here, the epidemiology, genotyping, and subtyping of *G. duodenalis* in humans and various other animals in China are summarized and reviewed.

## *G. duodenalis* in humans

Investigations and case reports on *G. duodenalis* infections in humans are common in China (Table 1). Sporadic reports of human giardiasis have been documented since 1962, although a number of giardiasis cases were recoded in 1983 in Xi'an (Zhang and Li, 1983). The large number of epidemiological investigations conducted at the start of this century suggested that the average infection rate was 0.85% (197/23,098), with the highest infection rate (9.46%, 7/74) reported by one study carried out in Shanghai (Wang L. et al., 2013). Differences in the observed rates of infection may be due, in part, to the age of the patients. In China, children <15 years of age were the most affected, with the peak infection rate occurring in those aged 5–10 years (Yu et al., 1994; Lv et al., 2013). A similar observation was made in Malaysia, where children under 15 years old were more likely to be infected with *G. duodenalis* (Mohammed Mahdy et al., 2009; Anuar et al., 2014).

**Table 1:** *Giardia duodenalis* infection rates and genotypes in humans in China.

**Locations**	**Patient group**	**Specimens**	**Positive (%)**	**Assemblage (no.)**	**Subassemblage (no.)**	**References**
Shaanxi:Xi'an	Patients	19^a^	Case reports			Zhang and Li, 1983
Anhui		10^a^	Genotypes identified	A (4)	AII (4)	Yong et al., 2000
				B (4)		
Sichuan	Diarrhea patients	2^a^	Case reports			Chen, 2001
Henan	Inpatients	18^a^	Genotypes identified	A (12)	AI(8); AII (4)	Wang et al., 2011
				B (6)		
Hebei: Chengde	Resident	216	3 (1.39%)	A (3)	AII (3)	Chen et al., 2000
Anhui: Huainan	School pupils	1,332	81 (6.08%)			Fu et al., 2004
Hainan: Haikou	Elementary school students	535	8 (1.50%)			Gan et al., 2006
Henan: Kaifeng	Patients	6,093	10 (0.16%)			Wang et al., 2009
Henan: Zhengzhou	Patients	4,836	11 (0.23%)			Sun et al., 2010
Henan: Zhengzhou	Children patients	1,996	12 (0.60%)			Xu et al., 2011
Anhui: Fuyang	HIV positive patients	302	4 (1.32%)			Tian et al., 2012
Anhui: Fuyang	HIV negative individuals	303	2 (0.66%)			Tian et al., 2012
Shanghai	Children with various congenital or inherited diseases	74^b^	7 (9.46%)	A (6)	AII (6)	Wang L. et al., 2013
Shanghai	Children attending the endocrinology	283	4 (1.41%)	A (2)	AII (2)	Wang L. et al., 2013
				B (2)		
Shanghai	children attending general surgeries	216	0			Wang L. et al., 2013
Shanghai	Children	3,472	25 (0.72%)	A (17)	AII (17)	Wang L. et al., 2013
				B (9)		
Shanghai	Diarrhea outpatients	252	17 (6.75%)	B (1)		Liu H. et al., 2014
				C (16)		
Hubei: Chibi	Kindergarten children	20	1 (5.00%)			Yuan et al., 2015
Shanghai	Diarrhea patients	95	1 (1.05%)	B (1)		Liu H. et al., 2015
Tibet: Lhasa	Resident	1,015	4 (0.39%)			Liu et al., 2016
Guangdong: Shenzhen	Diarrhea children	126	0			Shen et al., 2016
Guangdong: Shenzhen	Diarrhea adults patients	286	0			Shen et al., 2016
Guangdong: Shenzhen	Diarrhea patients <18	126	0			Shen et al., 2016
Yunnan: Kunming	Diarrhea children	850	0			Zhang S. X. et al., 2016
Yunnan: Kunming	Diarrhea children	170	0			Zhang S. X. et al., 2016
Wuhan	Diarrhea children	500	7 (1.40%)	A (7)	AII (7)	Wang T. et al., 2017
**Total**		**23,098**	**197 (0.85%)**	**A (51); B (23); C (16)**	**AI (8); AII (43)**	

a *Not included in the G. duodenalis infection rate calculation*.

b *Specimens from a cryptosporidiosis outbreak*.

Despite its widespread occurrence, molecular epidemiological data for *G. duodenalis* infections in humans from China is limited. According to the few available genotyping studies, both assemblage A (subtypes AI and AII) and B isolates have been found in China, with subtype AII and assemblage B being the dominant genotypes (Yong et al., 2000; Wang et al., 2011; Wang L. et al., 2013; Wang T. et al., 2017). Interestingly, a canid-specific assemblage C strain, which was first identified in Egypt (Soliman et al., 2011), was found in 16 *Giardia*-positive diarrheal outpatients in Shanghai (Liu H. et al., 2014). MLG analysis of assemblage AII and B isolates from Shanghai identified six and 11 sequence types, respectively (Wang L. et al., 2013). No significant gender-specific association for *G. duodenalis* infections or assemblage distribution has been reported in China (Liu H. et al., 2014; Wang T. et al., 2017).

## *G. duodenalis* in NHPs

The prevalence of *G. duodenalis* infections in NHPs varies markedly between different studies (Table 2). The average rate of infection for NHPs was 4.49% (172/3,827), with the highest rate recorded by a study conducted in Hunan Province (44.00%, 33/75). However, variability in the feeding habitats, health status, and age of the subjects, as well as differences in the geographic location and diagnostic techniques used in the studies probably contribute to the discrepant infection rates (Li J. et al., 2017).

**Table 2:** *Giardia duodenalis* infection rates and genotypes in non-human primates in China.

**Locations**	**Specimens**	**Positive (%)**	**Host species (no.)**	**Assemblage (no.)**	**Subassemblage (no.)**	**References**
Henan	74	1 (1.35%)	Rhesus macaque (1)			Zhao et al., 2011
Guangxi	232	0				Zhao et al., 2011
Sichuan	40	0				Zhao et al., 2011
Guizhou	411	35 (8.52%)	Rhesus macaque (10)	A (10)	AII (10)	Ye et al., 2012
			Rhesus macaque (24)	B (24)		
Guangxi	784	4 (0.51%)	Rhesus macaque / Cynomolgus monkey			Li J. et al., 2013
Guangxi	205	5 (2.44%)	Rhesus macaque (2)	A (2)	AII (2)	Ye et al., 2014
			Rhesus macaque (3)	B (3)		
Beijing	72	16 (22.22%)	Cynomolgus monkey (1)	A (1)	AIII (1)	Karim et al., 2014, 2015
			Ring-tailed lemur (6); Squirrel monkey (5); Golden monkey (3); Cynomolgus monkey (1)	B (15)	BIV (15)	
Hebei	89	10 (11.24%)	Ring-tailed lemur (5); Rhesus macaque (4); Mona monkey (1)	B (10)	BIV (10)	Karim et al., 2014, 2015
Henan	518	20 (3.86%)	Rhesus macaque (14); Japanese macaque (3); Olive baboon (2); Assam macaque (1)	B (20)	BIV (20)	Karim et al., 2014, 2015
Shanxi	66	9 (13.64%)	Rhesus macaque (5); Yellow baboon (2); Northern white-cheeked gibbon (2)	B (9)	BIV (9)	Karim et al., 2014, 2015
Shaanxi	197	4 (2.03%)	Rhesus macaque (3); Saimiri sciureus (1)	E (4)		Du et al., 2015
Shanghai	128	19 (14.84%)	Green monkey (1)	A (1)	AI (1)	Karim et al., 2014, 2015
			Ring-tailed lemur (10); Golden monkey (2); Squirrel monkey (2); Cynomolgus monkey (2) King colobus (1); Mandrill (1)	B (18)	BIV (18)	
Hubei	66	5 (7.58%)	Pig-tailed macaque (4); Hamadryas baboon (1)	B (5)	BIV (5)	Karim et al., 2014, 2015
Hunan	75	33 (44.00%)	Ring-tailed lemur (2)	A (2)	AI (1); AII (1)	Karim et al., 2014, 2015
			Pig-tailed macaque (8); Bornean orangutan (5); Hussar monkey (5); Ring-tailed lemur (3); Squirrel monkey (3); Cynomolgus monkey (3); Green monkey (2); Roloway monkey (1); Francois' leaf monkey (1)	B (31)	BIV (31)	
Guangdong	57	1 (1.75%)	Cynomolgus monkey (1)	B (1)	BIV (1)	Karim et al., 2014, 2015
Guangxi	363	9 (2.48%)	Rhesus macaque (8); White-headed (1)	B (9)	BIV (9)	Karim et al., 2014, 2015
Sichuan	304	0				Karim et al., 2014, 2015
Yunnan	144	0				Karim et al., 2014, 2015
Henan	2	1 (50.00%)	Nomascus leucogenys (1)	B (1)		Li J. et al., 2015
**Total**	**3,827**	**172 (4.49%)**		**A (16); B (146); E (4)**	**AI (2); AII (13); AIII (1); BIV (118)**	

Thus far, assemblage A, B, and E strains have been identified in NHPs, with assemblage B dominant in China (Karim et al., 2014, 2015). Only one study, from Shaanxi Province, identified an assemblage E isolate in NHPs in China (Du et al., 2015), although an assemblage E isolate has also been found in a red colobus monkey in western Uganda (Johnston et al., 2010). MLG has also been used for genotyping *G. duodenalis* in NHPs. Like humans, several assemblage A (subtypes AI and AII) and B (subtype BIV) subtypes have been identified in NHPs, with subtype BIV identified as the dominant subtype (Karim et al., 2014, 2015). A total of 15 MLG genotypes (two known and 13 novel) were reported in one study, although the two known MLG genotypes were not significant from a public health perspective (Karim et al., 2015).

Phylogenetic analysis has suggested the possibility of geographical segregation and host-adaptation amongst assemblage B strains in NHPs in China. The role of NHPs in the transmission of *G. duodenalis* to humans is not clear. It is believed that the frequent occurrence of assemblage B strains in captive NHPs may be associated with transmission from human sources, or an indication of adaptation to primate host (Sprong et al., 2009; Karim et al., 2015).

## *G. duodenalis* in cattle

In cattle, *G. duodenalis* infections vary in their prevalence and genotypic distribution according to region and cattle species (Table 3). The first documentation of *G. duodenalis* infection in dairy cattle occurred in 2006 in Guangdong Province (Xiao et al., 2006). The average infection rate in cattle (including dairy cattle, beef cattle, and yaks) is 5.43% (693/12,753), with the highest rate observed in Shaanxi Province (18.87%, 70/371). However, use of different detection methods may contribute to the observed differences in prevalence.

**Table 3:** *Giardia duodenalis* infection rates and genotypes in cattle in China.

**Animals**	**Locations**	**Specimens**	**Positive (%)**	**Assemblage (no.)**	**Subassemblage (no.)**	**References**
Dairy cattle	Guangdong	1^a^	Case report	E (1)		Xiao et al., 2006
Dairy cattle	Heilongjiang	26^a^	Genotypes identified	B (10)		Liu A. et al., 2014
				E (16)		
Dairy cattle	Jilin	249	19 (7.63%)	E (19)		Zhang J. et al., 2012
Dairy cattle	Heilongjiang	52	4 (7.69%)	A (1)	AI (1)	Zhang J. et al., 2012
				E (3)		
Dairy cattle	Heilongjiang	814	42 (5.16%)	B (18)	BI (6); BII (1); BIII (2); BIV (2); BV (1); BVI (1); BVII (1); BVIII (3); BIX (1)	Liu et al., 2012
				E (24)		
				A/E (1)		
Dairy cattle	Heilongjiang	52	8 (15.38%)	E (8)		Liu G. et al., 2015
Dairy cattle	Jilin	377	25 (6.63%)	A (1)	AI (1)	Liu G. et al., 2015
				E (24)		
Dairy cattle	Liaoning	226	19 (8.41%)	E (18)		Liu G. et al., 2015
				A/E (1)		
Dairy cattle	Beijing	822	14 (1.70%)	E (14)		Li F. et al., 2016
Dairy cattle	Henan	1,777	128 (7.20%)	A (21)	AI (4); AII (3); AIII (1)	Wang et al., 2014b
				E (58)		
				A/E (2)		
Dairy cattle	Henan	622	21 (3.38%)			Wang et al., 2014a
Dairy cattle	Henan	507	48 (9.47%)	E (48)		Wang C. et al., 2016
Dairy cattle	Henan	622	21 (3.38%)	E (21)		Zhao et al., 2016
Dairy cattle	Xinjiang	514	69 (13.42%)	A (5)	AI (3); AII (2)	Qi et al., 2016b
				E (64)		
Dairy cattle	Gansu	1,224	32 (2.61%)	E (32)		Zhang et al., 2016a
Dairy cattle	Ningxia	1,366	29 (2.12%)	B (4)	BI (1); BII (3)	Huang et al., 2014
				E (25)		
Dairy cattle	Ningxia	1,614	74 (4.58%)	A (1)		Zhang et al., 2016a
				E (73)		
Cattle	Qinghai	47	3 (6.38%)			Ma et al., 2014
Beef/dairy cattle	Shaanxi	371	70 (18.87%)	A (8)	AI (8)	Wang X. T. et al., 2016
				E (62)		
Yak	Qinghai	57	7 (12.28%)			Ma et al., 2014
Yak	Henan	34	2 (5.88%)	E (2)		Qi et al., 2015a
Yak	Gansu	117	4 (3.42%)	E (4)		Qi et al., 2015a
Yak	Sichuan	146	4 (2.74%)	E (4)		Qi et al., 2015a
Yak	Tibet	96	1 (1.04%)	E (1)		Qi et al., 2015a
Yak	Qinghai	152	5 (3.29%)	E (5)		Qi et al., 2015a
Yak	Qinghai	93	8 (8.60%)	E (9)		Wang et al., 2016a
Yak	Qinghai	297	22 (7.41%)	E (22)		Wang et al., 2016b
Yak	Gansu	208	4 (1.92%)	E (4)		Song et al., 2016
Yak	Qinghai	297	10 (3.37%)	E (10)		Wang G. et al., 2017
**Total**		**12,753**	**693 (5.43%)**	**E (571); A (37); B (32); A/E (4)**	**AI (17); AII (5); AIII (1); BI (7); BII (4); BIII (2); BIV (2); BV (1); BVI (1); BVII (1); BVIII (3); BIX (1)**	

a *Not included in the G. duodenalis infection rate calculation*.

There is a significant association between *G. duodenalis* infection and age in cattle. Most studies have reported that *G. duodenalis* infection rates are inversely associated with animal age in China (Liu et al., 2012; Huang et al., 2014; Wang et al., 2014b; Liu G. et al., 2015; Qi et al., 2015a; Li F. et al., 2016; Zhang et al., 2016b; Wang G. et al., 2017), except for a recent study from Xinjiang, which identified a higher prevalence in post-weaned calves (16.6%) compared with pre-weaned calves (9.7%; Qi et al., 2016b).

Cattle are dominantly infected with livestock-specific *G. duodenalis* assemblage E strains (Liu G. et al., 2015; Qi et al., 2015a; Li F. et al., 2016; Wang G. et al., 2017), with only a few reports of infection caused by assemblage A and/or B strains (Liu et al., 2012; Huang et al., 2014; Wang et al., 2014b; Zhang et al., 2016a). Moreover, sub-assemblages AI, AII, and AIII were identified by most studies conducted in China, with sub-assemblage AI found to be dominant (Wang et al., 2014b; Qi et al., 2016b; Wang X. T. et al., 2016). Mixed infections also appear to be common in cattle, especially those involving isolates belonging to assemblages A and E (Wang et al., 2014b; Liu G. et al., 2015).

Several studies using MLG have suggested the possibility of geographical distribution differentiation among assemblage E isolates in cattle (Wang et al., 2014b; Qi et al., 2016b; Wang X. T. et al., 2016; Zhang et al., 2016a). A MLG subtype AII isolate identical to human-derived isolates from Italy, Sweden, and China was identified in dairy cattle from Henan Province, raising the possibility of it being an important zoonotic multilocus genotype (Wang et al., 2014b).

Limited information is available on the prevalence and assemblage distribution of *G. duodenalis* in yaks, despite confirmed cases of infection in Qinghai, Gansu, Sichuan, and Henan Provinces, as well as in Tibet (Ma et al., 2014; Qi et al., 2015a; Song et al., 2016; Wang et al., 2016a,b; Wang G. et al., 2017). Thus far, only assemblage E isolates have been identified in yaks in China.

## *G. duodenalis* in sheep and goats

Reports of *G. duodenalis* infections in sheep and goats in recent years have presented variable results (Table 4). The average infection rate in sheep and goats is 6.07% (418/6,890), with the highest infection rate recorded in goats from Chongqing city (27.78%, 5/18). Except for one study that identified two assemblage B-type isolates in sheep in Heilongjiang (Zhang et al., 2012c), all reports of *G. duodenalis* infections in sheep and goats in China were caused by assemblage E and A strains, with assemblage E being significantly dominant (Gu et al., 2014; Peng et al., 2016; Wang H. et al., 2016). Mixed infections of assemblage A and E strains in sheep are commonly reported (Ye et al., 2015; Wang H. et al., 2016), while sub-assemblage AI was generally the dominant sub-genotype (Zhang et al., 2012c; Ma et al., 2014; Peng et al., 2016; Wang H. et al., 2016). A recent study from Henan Province using MLG yielded one new AI sub-assemblage with zoonotic potential, and six assemblage E MLGs (Wang H. et al., 2016).

**Table 4:** *Giardia duodenalis* infection rates and genotypes in sheep and goats in China.

**Animals**	**Locations**	**Specimens**	**Positive (%)**	**Assemblage (no.)**	**Subassemblage (no.)**	**References**
Sheep	Heilongjiang	21^a^	Genotypes identified	A (4)		Liu A. et al., 2014
				E (17)		
Sheep/Goat	Henan	880	16 (1.82%)			Sui et al., 2015
Sheep	Heilongjiang	539	25 (4.64%)	A (4)	AI(3), AIV(1)	Zhang et al., 2012c
				B (2)		
				E (19)		
Sheep	Henan	162	3 (1.85%)			Zhu et al., 2012
Sheep	Henan	1,028	58 (5.64%)			Li M. et al., 2013
Sheep	Henan	716	39 (5.45%)	A (5)	AI (9)	Wang H. et al., 2016
				E (31)		
				A/E (3)		
Sheep	Jilin	48	0			Li M. et al., 2013
Sheep	Liaoning	16	0			Li M. et al., 2013
Sheep	Shandong	17	0			Li M. et al., 2013
Sheep	Inner Mongolia	375	16 (4.27%)	A (13)	AI (4); AII (1); AIV (8)	Ye et al., 2015
				A/E (3)		
Sheep	Qinghai	61	8 (13.11%)			Ma et al., 2014
Goat	Heilongjiang	139	4 (2.88%)	E (4)		Zhang et al., 2012c
Goat	Henan	301	71 (23.59%)			Chen et al., 2015
Goat	Henan	63	1 (1.59%)			Li M. et al., 2012
Goat	Henan	844	48 (5.69%)			Zhu et al., 2013
Goat	Anhui	80	7 (8.75%)			Zhu et al., 2013
Goat	Chongqing	18	5 (27.78%)			Zhu et al., 2013
Goat	Qinghai	50	0			Zhu et al., 2013
Goat	Inner Mongolia	51	0			Zhu et al., 2013
Goat	Anhui	506	32 (6.32%)	E (32)		Gu et al., 2014
Goat	Qinghai	51	2 (3.92%)			Ma et al., 2014
Dairy goat	Henan	316	3 (0.95%)			Cao et al., 2015
Dairy goat	Shaanxi	170	11 (6.47%)	E (11)		Peng et al., 2016
Meat goat	Henan	144	35 (24.31%)	E (35)		Peng et al., 2016
Cashmere goat	Shaanxi	315	34 (10.79%)	A (4)	AIV (4)	Peng et al., 2016
				E (30)		
**Total**		**6,890**	**418 (6.07%)**	**E (179); A (30); B (2); A/E (6)**	**AI (16); AIV (13); AII (1);**	

a *Not included in the G. duodenalis infection rate calculation*.

## *G. duodenalis* in dogs and cats

In recent decades, a large number of cases of *G. duodenalis* infection in dogs, and some in cats, have been documented in different regions of China (Table 5). The first report of *G. duodenalis* infection in dogs occurred in 2000 in Jilin Province (He et al., 2000). The average infection rate in dogs is 13.64% (757/5,549), with the highest rate in Shanghai City (26.19%, 127/485) (Xu et al., 2016), whereas the average rate in cats is 10.19% (32/314), with the highest rate of infection also observed in Shanghai (13.13%, 21/160) (Xu et al., 2016).

**Table 5:** *Giardia duodenalis* infection rates and genotypes in dogs and cats in China.

**Animals**	**Locations**	**Specimens**	**Positive (%)**	**Assemblage (no.)**	**Subassemblage (no.)**	**References**
Dog	Jilin	1^a^	Case report			He et al., 2000
Dog	Jilin	1^a^	Case report			He et al., 2002
Dog	Beijing	2^a^	Case reports			Gao et al., 2009
Dog	Guangdong	1^a^	Case report	A (1)		Zhu et al., 2011
Dog	Guangdong	2^a^	Case reports	A (1)	AI (1)	Zhang et al., 2011
				D (1)		
Dog	Guangdong	1^a^	Case report	D (1)		Li et al., 2011
Dog	Guangdong	1^a^	Case report	D (1)		Li et al., 2012a
Dog	Shaanxi	56^a^	Case statistics			Quan et al., 2016
Dog	Jinlin	242	61 (25.21%)			He et al., 2001
Dog	Henan	404	15 (3.71%)			Qi et al., 2010a
Dog	Henan	531	72 (13.56%)			Qi et al., 2011
Dog	Sichuan	146	46 (31.51%)			Hu et al., 2011
Dog	Beijing	910	109 (11.98%)			Bi et al., 2011
Dog	Shaanxi	120	11 (9.17%)			Wang et al., 2015
Dog	Henan	358	71 (19. 83%)			Dong et al., 2015
Dog	Guangdong	209	23 (11.00%)	A (5)	AI (5)	Li et al., 2012b
				D (23)		
Dog	Liaoning	205	27 (13.17%)	A (25)	AI (25)	Li W. et al., 2013
				C (2)		
Dog	Guangdong	216	21 (9.72%)	A (7)	AI (7)	Zheng et al., 2014
				C (2)		
				D (1)		
				A/C (2);		
				A/D (7);		
				C/D (2)		
Dog	Heilongjiang	267	12 (4.49%)	C (7)		Li W. et al., 2015
				E (5)		
Dog	Henan	940	134 (14.26%)	C (37)		Qi et al., 2016a
				D (44)		
Dog	Shanghai	485	127 (26.19%)	A (23)	AII (23)	Xu et al., 2016
				B (1)		
				C (26)		
				D (58)		
				A/C (2)		
				A/D (1)		
				C/D (10)		
Dog	Qinghai	31	2 (6.45%)			Ma et al., 2014
Dog	Qinghai	10	1 (10.00%)	A (1)		Wang G. et al., 2013
Dog	Taiwan	42	4 (9.52%)			Liang et al., 2012
Dog	Taiwan	118	11 (9.32%)	C (7)		Tseng et al., 2014
				D (4)		
						
Dog	Anhui	215	10 (4.65%)	B (6)		Gu et al., 2015
				D (4)		
Dog	Zhejiang	100	0			Gu et al., 2015
**Subtotal**		**5,549**	**757 (13.64%)**	**D (137); C (81); A (63); B (7); E (5); C/D (12); A/D (8); A/C (4)**	**AI (38); AII (23)**	
Cat	Hebei	1^a^	Case report			Cui et al., 2010
Cat	Guangdong	1^a^	Case report	F (1)		Zheng et al., 2013
Cat	Guangdong	102	10 (9.80%)	A (8)	AI (8)	Zheng et al., 2015
				F (1)		
				A/C (1)		
Cat	Shanghai	160	21 (13.13%)	A (2)	AI (1); AII (1)	Xu et al., 2016
				B (6);		
				C (2)		
				D (1)		
				F (7)		
Cat	Heilongjiang	52	1 (1.92%)	F (1)		Li W. et al., 2015
**Subtotal**		**314**	**32 (10.19%)**	**A (10); F (10); B (6); C (2); D (1); A/C (1)**	**AI (9); AII (1)**	

a *Not included in the G. duodenalis infection rate calculation*.

Scant information on the epidemiology or molecular characteristics of *G. duodenalis* in dogs and cats in China is currently available. *G. duodenalis* assemblages A, C, and D have been identified as the most common genotypes in dogs, with A and F most prevalent in cats. Occasionally, assemblage B and E isolates have been reported (e.g., in two studies on dogs; Gu et al., 2015; Li W. et al., 2015), while assemblage B, C, and D isolates have been reported in cats (Zheng et al., 2015; Xu et al., 2016).

In general, sub-assemblage AI appears to be the dominant sub-genotype amongst isolates derived from dogs and cats in China (Li et al., 2012b; Li W. et al., 2013; Zheng et al., 2014, 2015), which agrees with findings from the limited number of reports from dogs and cats in Europe, USA, Brazil, Australia, and Japan (Vasilopulos et al., 2007; Volotão et al., 2007; Sprong et al., 2009; Feng and Xiao, 2011). However, one study from Shanghai showed that amongst 25 assemblage A sequences obtained from dog and cat specimens, 23 canine sequences and one feline sequence were identified as subtype AII (Xu et al., 2016).

## *G. duodenalis* in pigs

*G. duodenalis* infections in pigs have been reported in Australia, Africa, Asia, Europe, and North America (Feng and Xiao, 2011). However, there are limited reports on the prevalence and genotypes of this organism in pigs in China, except in Sichuan Province, where the average infection rate was 3.51% (24/683, Table 6). Although assemblage E was the predominant genotype amongst these isolates from China, assemblage A is also frequently identified (Li W. et al., 2016; Li J. et al., 2017). In contrast, isolates belonging to assemblages A–F have been found in domestic pigs in other countries, with assemblage B and E isolates found in Canada (Budu-Amoako et al., 2012), assemblage C and F isolates in the UK (Minetti et al., 2014), assemblage A, D, and E isolates in Denmark (Petersen et al., 2015), and isolates belonging to assemblages A, E, and F identified in Australia (Armson et al., 2009). Thus far, the majority of the assemblage A strains from pigs in China belong to sub-assemblage AI (Li W. et al., 2016, 2017).

**Table 6:** *Giardia duodenalis* infection rates and genotypes in pigs, rabbits, and rodents in China.

**Animals**	**Locations**	**Specimens**	**Positive (%)**	**Assemblage (no.)**	**Subassemblage (no.)**	**References**
Wild boars	Sichuan	308	11 (3.57%)	A (1)	AI (1)	Li W. et al., 2016
				E (10)		
Pig	Sichuan	18	2 (11.11%)	E (2)		Li W. et al., 2016
Wild boars	Sichuan	357	11 (3.08%)	A (2)	AI (2)	Li J. et al., 2017
				E (9)		
**Subtotal**		**683**	**24 (3.51%)**	**E (21); A (3)**	**AI (3)**	
Rabbits	Heilongjiang	14^a^	Genotypes identified	B (14)		Liu A. et al., 2014
Rabbits	Henan	1,027	80 (7.79%)			Xi et al., 2011b
Rabbits	Henan	1,081	57 (5.27%)			Shi et al., 2010
Rabbits	Henan	305	12 (3.93%)			Xi et al., 2011a
Rabbits	Heilongjiang	378	28 (7.40%)	B (28)	BI (18);BII (4); BIII (3); BIV (1); BV (1); BVI (1)	Zhang et al., 2012b
Rabbits	Henan	955	80 (8.38%)	B (26)	BIV (26)	Qi et al., 2015b
				E (2)		
				B/E (4)		
**Subtotal**		**3,746**	**257 (6.86%)**	**B (68); E (2); B/E (4)**	**BI (18); BII (4); BIII (3); BIV (27); BV (1); BVI (1)**	
Rodent	Henan	140	38 (27.14%)	A (5)	AI (5)	Qi et al., 2015c
				B (33)	BIV (31)	
Rodent	Henan	232	14 (6.03%)	G (14)		Zhao Z. et al., 2015
Rodent	Henan	96	36 (37.50%)			Lv et al., 2009b
Rodent	Henan	439	91 (20.72%)			Qi et al., 2010b
Rodent	Henan	153	34 (22.22%)			Lv et al., 2009a
**Subtotal**		**1,060**	**213 (20.09%)**	**B (33); G (14); A (5)**	**AI (5); BIV(31)**	

a *Not included in the G. duodenalis infection rate calculation*.

## *G. duodenalis* in rabbits

*G. duodenalis* infections occur in rabbits in China at an average rate of 6.86% (271/3,746), and have mainly been documented in Henan and Heilongjiang Provinces (Table 6). Although assemblage E isolates are occasionally found (Qi et al., 2015b), assemblage B strains appear dominant in rabbits in China (Zhang et al., 2012b; Liu A. et al., 2014; Qi et al., 2015b), which agrees with reports from Europe (Pantchev et al., 2014) and the USA (Sulaiman et al., 2003).

## *G. duodenalis* in rodents

*G. duodenalis* infections have been reported in rodents in Norway (Robertson et al., 2007), Poland (Bajer, 2008), Latin America (Bueno et al., 2016), Europe (Pantchev et al., 2014), and Sweden (Lebbad et al., 2010). Currently, *G. duodenalis* infections in rodents in China have only been reported in Henan Province, where the average infection rate was 20.09% (213/1,060; Table 6). According to the limited number of genotyping studies on *G. duodenalis* in rodents in China, only assemblage A and B strains, which have zoonotic potential, and host-adapted assemblage G isolates have been identified (Qi et al., 2015c; Zhao Z. et al., 2015).

## *G. duodenalis* in other mammals

*G. duodenalis* infections have also been reported in beavers, Chinese leopards, Siberian tigers, golden takins, raccoon dogs, horses, deer, and donkeys (Table 7). Some of these infections have high prevalence rates, such as in donkeys in Shandong Province (18.27%, 19/104) (Zhang et al., 2017), raccoon dogs in Liaoning Province (15.28%, 11/72) (Zhang et al., 2016b), golden takins in Shaanxi Province (8.90%, 17/191) (Zhao G. H. et al., 2015), and Pika (9.09%, 1/11) and donkeys (7.69%, 1/13) in Qinghai Province (Ma et al., 2014).

**Table 7:** *Giardia duodenalis* infection rates and genotypes in other mammals in China.

**Animals**	**Locations**	**Specimens**	**Positive (%)**	**Assemblage (no.)**	**References**
Pika	Qinghai	11	1 (9.09%)		Ma et al., 2014
Chinese leopard	Henan	2	1 (50.00%)	F (1)	Li J. et al., 2015
Beaver	Henan	1	1 (100.00%)	B (1)	Li J. et al., 2015
Siberian tiger	Henan	6	2 (33.33%)	F (2)	Li J. et al., 2015
Golden takins	Shaanxi	191	17 (8.90%)	B (3)	Zhao G. H. et al., 2015
				E (14)	
Grazing horses	Xinjiang	262	4 (1.50%)	A (2)	Qi et al., 2015d
				B (2)	
Raccoon dog	Jilin	110	7 (6.36%)	C (6)	Zhang et al., 2016b
				C/D (1)	
Raccoon dog	Heilongjiang	40	3 (7.50%)	C (3)	Zhang et al., 2016b
Raccoon dog	Shandong	29	0		Zhang et al., 2016b
Raccoon dog	Hebei	54	1 (1.85%)	C (1)	Zhang et al., 2016b
Raccoon dog	Liaoning	72	11 (15.28%)	C (10)	Zhang et al., 2016b
				C/D (1)	
Donkey	Qinghai	13	1 (7.69%)		Ma et al., 2014
Donkey	Jilin	48	5 (10.42%)	B (5)	Zhang et al., 2017
Donkey	Shandong	104	19 (18.27%)	B (19)	Zhang et al., 2017
Donkey	Liaoningz	29	4 (13.79%)	B (4)	Zhang et al., 2017
Deer	Henan	199	5 (2.51%)	E (5)	Unpublished
**Total**		**1,171**	**82 (7.00%)**	**A (2); B (34); C (20); E (19); F (3); C/D (2)**	

Certain *G. duodenalis* assemblages have been associated with host adaptation in specific animals, such as, assemblages C and D in raccoon dogs (Zhang et al., 2016b), assemblage E in golden takins (Zhao G. H. et al., 2015) and deer (unpublished data), and assemblage F in a Chinese leopard and Siberian tigers (Li J. et al., 2015). Zoonotic *G. duodenalis* isolates belonging to assemblages A and B were also identified in beavers (Li J. et al., 2015), golden takins (Zhao G. H. et al., 2015), and horses (Qi et al., 2015d). The number of zoonotic isolates found in wild animals is quite limited, an observation supported by a large survey of *G. duodenalis* in wild mammals from Croatia that revealed a low prevalence and limited zoonotic potential for the parasite (Beck et al., 2011). This suggests that wild animals are probably not a major reservoir for human infections.

## *G. duodenalis* in wastewater

Cases of giardiasis associated with polluted recreational and potable waters have been documented for more than a century worldwide (Guy et al., 2003; Karanis et al., 2007; Moss, 2016). Although there have been no *G. duodenalis* outbreaks documented in China, a high prevalence of oocysts was identified amongst samples from numerous municipal and domestic raw water sources in Shanghai (Zhang et al., 2010; Li N. et al., 2012), Guangzhou (Zhong et al., 2010; Sun et al., 2014), Wuhan (Li N. et al., 2012; Sun et al., 2014), Jiangsu (Sun et al., 2014), Harbin (Liu et al., 2011; Zhang et al., 2012a), Guiyang (Chen et al., 2009), Nanjing (Li N. et al., 2012), Qingdao (Li N. et al., 2012), Taiwan (Liang et al., 2012), and Qinghai (Ma et al., 2014), among others.

Assemblage A and B isolates, which have zoonotic potential, were identified in urban waste in China (Liu et al., 2011; Liang et al., 2012; Li N. et al., 2012), suggesting that this pathogen could be maintained and transmitted by water sources, with the attendant risk of disease outbreaks occurring. Similarly, zoonotic isolates were also identified in Iran (Mahmoudi et al., 2015), Australia (Nolan et al., 2013; Koehler et al., 2016), Malaysia (Lim et al., 2009a,b, and Portugal (Lobo et al., 2009), among others. Isolates belonging to other assemblages, such as assemblage E, were also documented in wastewater in France (Bertrand and Schwartzbrod, 2007).

## Conclusions and perspectives

In conclusion, *G. duodenalis* is widely distributed in humans and various other animals in China. Among the *G. duodenalis* assemblages, A and B are considered to have the broadest host specificities, and strains belonging to these assemblage types have zoonotic potential. Generally speaking, assemblage A isolates are more frequently found in humans, livestock, and companion animals, while assemblage B isolates are commonly isolated from humans, NHPs, and rabbits, with only a few reports of infections in sheep, goats, dogs, and cats in China. Cattle, sheep, goats, and pigs are predominantly infected with host-specific assemblage E isolates, while assemblage C and D isolates have been found in dogs, assemblage F is associated with cats, and rodents tend to be infected with assemblage G isolates. Within assemblage A, humans and NHPs are more commonly infected with subgroup AII isolates, while in other animals, sub-assemblage AI is predominant.

Most molecular investigations of *G. duodenalis* in China have only examined one or two loci, which cannot provide sufficient information on the transmission profile of this pathogen. However, the availability of the whole genome sequence of *G. duodenalis* has enhanced population genetics-based studies, and multi-locus sequence typing (MLST) tools are increasingly being used for characterizing *G. duodenalis* infections in humans and animals. Access to these methods is especially important for gaining a better understanding of some host-adapted assemblages (e.g., C, D, E, and F) that are pathogenic in humans, and for assessing the zoonotic assemblages (A and B) with infective potential. Therefore, a comprehensive and systematic study based on MLST analysis should be carried out to allow a full assessment of the burden of giardiasis of animal origin in humans.

## Author contributions

LZ conceived the idea for the review and revised the manuscript. JL and HW wrote the manuscript, and JL, HW, and RW reviewed and abstracted the data from each selected article.

### Conflict of interest statement

The authors declare that the research was conducted in the absence of any commercial or financial relationships that could be construed as a potential conflict of interest.
